# Socioeconomic determinants affecting the access and utilization of depression care services in immigrants: A population-based study

**DOI:** 10.1371/journal.pone.0213020

**Published:** 2019-03-13

**Authors:** Sohyun Jeong, Cinoo Kang, Hyemin Cho, Hee-Jin Kang, Sunmee Jang

**Affiliations:** 1 College of Pharmacy and Gachon Institute of Pharmaceutical Sciences, Gachon University, Incheon, Korea; 2 Big Data Steering Department of National Health Insurance Service, Wonju, Korea; University of the West Indies Faculty of Medical Sciences Mona, JAMAICA

## Abstract

**Background:**

It is imperative to address the health problems faced by immigrants in their destination countries in light of the current magnitude of migration processes worldwide. We aimed to evaluate the socioeconomic determinants of healthcare utilization in immigrants with depression.

**Method:**

A population-based cohort comprising all immigrants who were eligible for National Health Insurance coverage (permanent residents, marriage immigrants, and naturalized citizens) using the National Health Insurance Claims Database in 2011–2013 was established. Cases were defined as immigrants with new-onset depression. Controls were new-onset Korean patients with depression matched by age, sex, and Charlson comorbidity index in a 1:2 ratio. Appropriateness of care (AOC) was defined as visiting a clinic for depression management at least 3 times in the first 12 weeks and 4 times thereafter until 12 months post-cohort entry.

**Results:**

A total of 2,378 immigrants and 4,756 matched Korean patients were identified. Of the immigrants, 30.0% achieved AOC, in contrast to 38.7% of Koreans (p < .0001). Adjusting for possible covariates, AOC was less likely for immigrants (adjusted OR (aOR), 0.760; 95% CI: 0.670–0.863). Medical Aid (aOR, 2.309; 95% CI, 1.479–3.610), rural residence (aOR, 1.536; 95% CI, 1.054–2.237), the presence of a psychiatric comorbidity (aOR, 1.912; 95% CI, 1.484–2.463), and visiting a psychiatrist (aOR, 2.387; 95% CI, 1.821–3.125) were associated with an increased likelihood of AOC in immigrants.

**Conclusion:**

Socioeconomic determinants included insurance type (Medical Aid and National Health Insurance), place of residence, psychiatric comorbid status, doctor specialty, easy access to medical services (clinic-based), and a SSRI-based treatment regimen. Those predictors should be taken into account when developing healthcare strategies for immigrants.

## Introduction

In the current age of rapid globalization, immigration has exerted significant effects on nations and people globally [1]. The number of international migrants has consistently increased, reaching 258 million in 2017 (a 40% increase compared to the number of 173 million in 2000), and over 60% of international migrants reside in Asia (80 million) and Europe (78 million) [2]. The number of immigrants in Korea reached 2 million in 2016, accounting for 4.0% of the Korean population [3], which reflects a rapid doubling from 2007 [3, 4]. The main country of origin of immigrants in Korea is China, followed by Vietnam and Thailand [3]. The steep increase of immigration to Korea began in the late 1980s, with labor immigration accounting for the majority of migrants, followed by marriage immigration starting in the early 1990s and the immigration of international students starting in 2015; as a result, Korea has become a highly diverse society. The proportion of immigrants and their offspring was 3.4% of the total Korean population in 2015. The Korean government has been providing language education, family consultations, cultural adaptation programs, and diverse policies to support immigrants and to promote their smooth assimilation into Korean society [5].

International migration has offered new opportunities for millions of people worldwide, although it also poses multiple issues concerning economic, social, security, and health affairs [1]. Immigration is a complex process generally regarded as a stressful life event [6]. In general, according to the “healthy immigrant” theory, immigrants have comparable health conditions to native residents in the early immigration period. However, this pattern reverses in the middle to late immigration period because of the hardships that immigrants overcome and the underutilization of health care services in the process of assimilation to their new country [7, 8].

According to a recent meta-analysis comprising 21 studies [9], immigrants are more prone to depression than native residents. Some studies reported a high tendency for immigrants to be vulnerable to depression, anxiety disorders, and somatization [10, 11]. This may be due to the traumatic past experiences that forced people to leave their home countries, the distress faced during assimilation to new environment, or their separation from close family members and social relationships in their countries of origin [12].

Major depressive disorder (MDD) is a common, recurrent psychiatric disease leading to diminished life functioning and quality of life. MDD patients are also at an increased risk for developing other psychiatric and medical disorders as complications [13], as well as several other chronic diseases, including coronary heart disease [14] and diabetes [15]. The World Health Organization has ranked depression as the fourth leading cause of disability worldwide [16] and has predicted that it will become the second leading cause of disability by 2020 [17].

Preventing and managing common mental health problems can be challenging for immigrants because of low literacy [18–20] and cultural gaps in ways and perceptions of seeking medical help [21–23]. Since poor mental health is associated with social isolation, unemployment and increased risk of suicide [24], and depressed patients are 1.76 times more likely not to adhere to treatment than nondepressed patients, as proven in a recent meta-analysis [25], targeting depressed patients as a high-risk group for medication non-adherence might be the standard of care [25–27].

Recently, the Korean Ministry of Health and Welfare revised the provisions regarding National Health Insurance coverage for immigrants. Immigrants who reside in Korea for more than 6 months are encouraged to enroll in National Health Insurance, and if they do so, they are eligible for the same benefits as Korean citizens. In 2017, 59.4% of immigrants had enrolled, which is approximately below half of the proportion of Korean citizens who were covered (95.6%)[28].

Therefore, in this context, an evaluation of the social, economic, and clinical factors affecting the consistency of depression treatment would provide insights into the management of other health problems and ways to establish a robust health care strategy for immigrants [29].

Considering the lack of population-based research on mental health among immigrants, we aimed to assess the factors affecting appropriate health care utilization among immigrants for depression management using the National Health Insurance Database.

## Methods

### Database

A database comprising all immigrants who were eligible for national health insurance coverage (permanent residents, marriage immigrants, and naturalized citizens) using the National Health Insurance Claims Database combined with pharmacy prescription claim data in 2011–2013 was established; the year 2011 was used for the screening period, 2012 for enrollment, and 2013 for the follow-up period. The national health insurance program in Korea provides universal coverage for the entire population, of approximately 50 million Koreans, so healthcare utilization by immigrants who are eligible for national insurance coverage can be analyzed systematically. The database contains all information on diagnoses, prescribed drug names, prescription duration, ambulatory visits, and hospitalization.

This study was approved by Gachon University Institutional Review Board (IRB No. 1044396-201710-HR-169-01). Although this research involved individual participants’ healthcare insurance claims data, we acquired the data in a form in which individual identities had been deleted (secondary data), and the data only contained items pertaining to this research purpose. Therefore, the IRB did not require us to obtain informed consent from individual patients.

All procedures performed in studies involving human participants were conducted in accordance with the ethical standards of the institutional and/or national research committee and with the 1964 Helsinki Declaration and its later amendments or comparable ethical standards.

### Study design and definition of subjects

A 1-year case-controlled study design was used, with the goal of observing and assessing the socioeconomic determinants of immigrants with depressive disorder in comparison with those of Koreans in the context of health care service accessibility and appropriateness.

Case subjects were defined as immigrants who were eligible for national healthcare insurance coverage and 1) were 20–100 years of age, 2) had a diagnosis code for depressive disorder (KCD-10: F32, F33, F34.1, F41.2), 3) had an antidepressant prescription filled at the time of the first diagnosis and visited a clinic at least twice during the follow-up period, and 4) were able to be followed up for 1 year.

To include pure incident cases, we excluded patients who had a diagnosis code of depression or filled a prescription for antidepressants in the 6-month period before cohort entry (screening period). Since appropriateness of care (AOC) was assessed on the basis of the frequency of outpatient clinic visits, we also excluded patients who were ever hospitalized for any cause during the follow-up period.

The index date (cohort entry date) for cases was defined as the date of the first antidepressant prescription filled for depressive disorder in 2012. Controls were defined using the same inclusion and exclusion criteria, applied to Korean depression patients. Up to 2 controls were matched to each case by sex, age, and Charlson comorbidity index (CCI) score. The index date for controls was defined using the same criteria as for the case group: the first antidepressant prescription filled for depressive disorder in 2012.

### Definition of AOC in depression management

According to the clinical guidelines of Korea [30] and an article on depression management in the US [31], depression management was defined as appropriate when patients visited a clinic for depression management at least 3 times in the first 12 weeks after the first antidepressant prescription was filled and 4 times thereafter until 12 months. AOC for depression management should include at least 7 visits per year.

### Statistical analysis

Patient characteristics at cohort entry were presented as counts with percentages for categorical data and means with standard deviations for continuous data. Age, sex, CCI scores, residential region, insurance type (Medical Aid and National Health Insurance [NHI]; Medical Aid: full coverage by national health insurance; NHI: approximately 70% coverage for all medical expenditures, with patients paying for 30% of expenditures out of pocket), type of medical institution, psychiatric comorbidities, number of household members, and the specialty of the doctor who was seen were analyzed.

The antidepressants prescribed at the first diagnosis were categorized as selective serotonin reuptake inhibitors (SSRIs), tricyclic antidepressants (TCAs), and SSRI + others (SSRI treatment with another co-medication for depression), and others, as appropriate in light of the prevalent prescription trends in Korea identified in this research. Outpatient visits in the acute and continuation periods were tested by the Wilcoxon signed-rank test to evaluate differences in AOC achievement because the data used did not satisfy the assumption of a normal distribution for the Kolmogorov-Smirnov and Shapiro-Wilk tests.

Logistic multivariate regression was performed to compare AOC in depression management between immigrants and native Koreans, and to identify socioeconomic and medical predictors affecting immigrants’ AOC. The odds ratios (ORs) for AOC in depression management and the corresponding 95% CIs were estimated, adjusting for possible confounders (covariates that were statistically significantly different at baseline). All analyses were performed using SAS version 9.4 (SAS Institute, Cary, NC, USA).

## Results

### Study population

**Fig 1:**
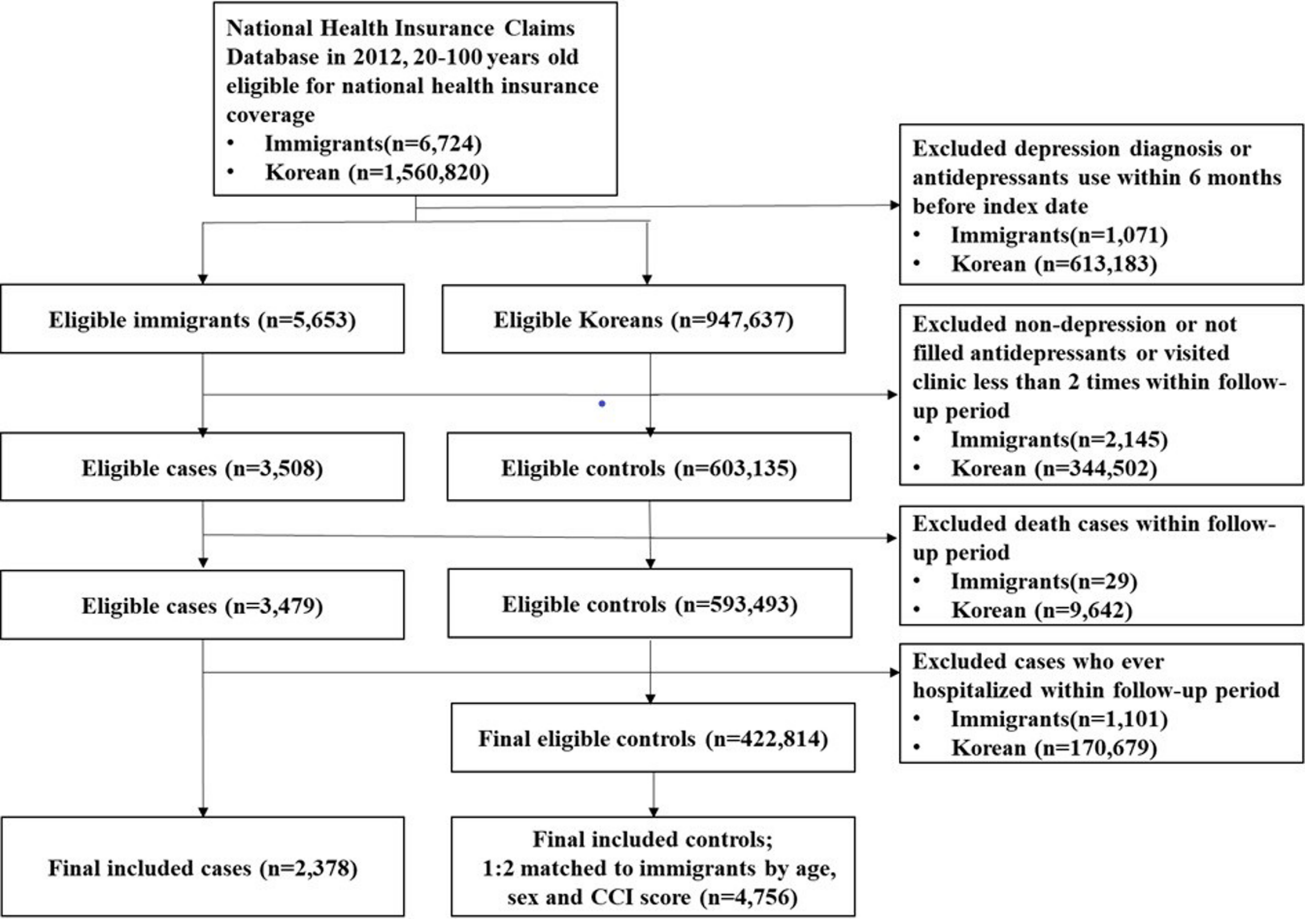
The selection process of study subjects; incident depression patients of immigrants and Korean.

A total of 2,378 immigrants with depression who satisfied the case definitions and 4,756 Korean controls with depression who were randomly matched by age, sex, and CCI index to cases at a 1:2 ratio were identified (Fig 1).

The demographic characteristics of the study population are presented and compared in Table 1. Age, sex, and CCI were matched between cases and controls. No notable differences were found in residential region, type of insurance, or number of antidepressants. However, the type of clinic for depression management, specialty of the doctor who was seen, psychiatric comorbidities, and type of antidepressants prescribed at the first visit were significantly different between cases and controls.

**Table 1:** Characteristics of immigrants and native Koreans with depression.

Characteristics		All		Immigrants/Koreans				p-value
				Immigrants		Koreans		
		N	%	N	%	N	%	
Categories		7,134	100	2,378	100	4,756	100	
Sex	Male	1,183	16.6	396	16.7	787	16.5	0.9104
	Female	5,951	83.4	1,982	83.3	3,969	83.5	.
Age	[mean, ±SD]	47.66	13.79	47.68	13.85	47.64	13.77	
	20~40 y	2,214	31	741	31.2	1,473	31	0.9258
	40~64 y	4,035	56.6	1,338	56.3	2,697	56.7	.
	65 y and over	885	12.4	299	12.6	586	12.3	.
CCI	[mean, ±SD]	0.61	0.93	0.61	0.94	0.61	0.92	
	0	4,248	59.5	1,421	59.8	2,827	59.4	0.9437
	1	1,911	26.8	631	26.5	1,280	26.9	.
	2 and over	975	13.7	326	13.7	649	13.6	.
Residential region	Urban site	6,530	91.5	2,196	92.3	4,334	91.1	0.0811
	Rural site	604	8.5	182	7.7	422	8.9	.
Type of insurance	NHI	6,692	93.8	2,219	93.3	4,473	94	0.2242
	Medical aid	442	6.2	159	6.7	283	6	.
Type of clinic for depression management	Tertiary hospital	2,274	31.9	854	35.9	1,420	29.9	< .0001
	Secondary hospital	805	11.3	279	11.7	526	11.1	.
	Clinic	4,055	56.8	1,245	52.4	2,810	59.1	.
Psychiatric comorbidities*	No	5,647	79.2	1,993	83.8	3,654	76.8	< .001
	Yes	1,487	20.8	385	16.2	1,102	23.2	.
Number of household members	[mean, ±SD]	2.84	1.58	2.5	1.45	3.02	1.61	
	1	1,821	25.5	718	30.2	1,103	23.2	< .001
	2–3	2,975	41.7	1,113	46.8	1,862	39.2	.
	4 and over	2,338	32.8	547	23	1,791	37.7	.
Doctor specialty	Psychiatry	3,750	52.6	1,376	57.9	2,374	49.9	< .001
	Others	3,384	47.4	1,002	42.1	2,382	50.1	.
Antidepressant prescribed at the first visit	(single) TCA	2,106	29.5	760	32	1,346	28.3	< .001
	(single) SSRI	2,213	31	639	26.9	1,574	33.1	.
	(complex) SSRI plus others#	565	7.9	197	8.3	368	7.7	.
	Others##	2,250	31.5	782	32.9	1,468	30.9	.
Number of antidepressants	[mean, ±SD]	1.12	0.36	1.11	0.33	1.13	0.37	
	1	6,321	88.6	2,123	89.3	4,198	88.3	0.206
	2 and over	813	11.4	255	10.7	558	11.7	.
Type of immigration	Permanent resident			405	17.03			
	Marriage immigrant			502	21.11			
	Naturalized citizen			1471	61.86			
Country of origin	China			683	28.72			
	Taiwan			64	2.69			
	Japan			41	1.72			
	others			118	4.96			
	N/A			1472	61.91			

*Psychiatric comorbidities included: psychotic disorder, manic diseases, bipolar, other affective disorders, anxiety, eating disorder, sleep disorder, personality disorder, compulsive behavior disorder.

#SSRI plus other co-medications for depression treatment

##MAOIs (Monoamine oxidase inhibitors), SNRIs(serotonin-norepinephrine reuptake inhibitors), NDRIs (norepinephrine–dopamine reuptake inhibitors), NARIs(Noradrenergic reuptake inhibitors), NaSSAs(noradrenergic and specific serotonergic antidepressants), SARIs (Serotonin antagonist and reuptake inhibitors), etc.

Among the immigrants, female immigrants (83.3%), the age group of 40–64 years (56.3%), and the group with comparable CCIs to the normal population (CCI score 0, 59.8%) were predominant. Urban residence was prevalent (92.3%) and the Medical Aid coverage rate was 6.7%.

Among the various baseline characteristics, immigrants were more likely to use tertiary hospitals (clinical centers for foreigners tend to be located in tertiary hospitals), to have fewer household members, to visit psychiatrists more often, and to have fewer prescriptions of SSRIs than of other antidepressants.

Among the immigrants, 61.86% were naturalized, followed by marriage immigrants (21.11%) and permanent residents (17.03%). China was the most common country of origin (28.72%), followed by Taiwan (2.69%) and Japan (1.72%). However, when immigrants are naturalized, their country of origin in the database is changed to Korea, so exact information on their country of origin was not available. This was the case for 61.86% of the sample, and was reflected by “N/A” being the most common result for country of origin (61.91%). The N/A option (1,472) for country of origin was observed for 6 permanent residents, 1 marriage immigrant, and 1,465 naturalized citizens (Table 1).

### AOC in depression management in immigrants compared to native Korean

During the 1-year follow-up, the immigrants achieved less AOC for depression management; the mean number of clinic visits for the immigrants was 6.28 (6.37), while it was 7.77 (7.91) for Koreans (*p <* .0001). Among the immigrants, 30.0% achieved AOC in depression management (7 or more clinical visits), whereas 38.7% of Korean depressed patients did so (*p <* .0001).

In the acute phase, the immigrants made fewer clinic visits than the Koreans [3.56 (2.65) vs. 3.87 (3.01)] (*p <* .0001); 54.0% of the immigrants satisfied AOC (3 or more clinical visits) while 56.8% of Koreans did so (*p =* .0002). In the continuation period, the immigrants made fewer clinic visits than the Koreans [4.95 (5.34) vs. 6.15 (6.4)] (*p <* .0001); during this period, 25.2% of the immigrants satisfied AOC (4 or more clinical visits), in contrast to 33.7% of Koreans (*p <* .0001) (Table 2).

**Table 2:** The number of outpatient visits in the acute and continuation period (1 year follow up) and the proportion of immigrants and Koreans who achieved AOC^#^ for depression management.

		Total		Subjects (N = 7,134)				p-value*
				Immigrants (N = 2,378)		Koreans (N = 4,756)		
		n	%	n	%	n	%	
1 year follow-up	Total visits, n	51,916		14,941		36,975		< .0001
	Visits [mean, ±SD]	7.28±7.47		6.28±6.37		7.77±7.91		
	< 7	4,582	64.2	1,665	70	2,917	61.3	
	> = 7	2,552	35.8	713	30	1,839	38.7	
Within 12 weeks (acute phase)	Total visits, n	26,898		8,472		18,426		< .0001
	Visits [mean, ±SD]	3.77±2.9		3.56±2.65		3.87±3.01		
	<3	3,147	44.1	1,093	46	2,054	43.2	
	> = 3	3,987	55.9	1,285	54	2,702	56.8	
13 weeks to 12 months (continuation phase)	Total visits, n	24,994		6,468		18,526		< .0001
	Visits [mean, ±SD]	5.79±6.12		4.95±5.34		6.15±6.4		
	<4	4,930	69.1	1,779	74.8	3,151	66.3	
	> = 4	2,204	30.9	599	25.2	1,605	33.7	

*Tested by the non-parametric Wilcoxon signed-rank test.

# AOC, appropriateness of care.

We observed that AOC was less achieved in the immigrants than in native Koreans with depression by multivariate logistic regression. The overall likelihood of AOC was 0.679 times lower in the immigrants (OR, 0.679; 95% CI, 0.611–0.755). When adjusting for type of insurance, residential region, type of clinic most often visited, comorbid psychiatric disease, number of household members, specialty of the doctor who was seen, and the antidepressant prescribed at the first visit, the OR for AOC increased (aOR, 0.761; 95% CI: 0.670–0.863) (Table 3).

**Table 3:** Appropriateness of care (AOC) in depression management in immigrants compared to native Koreans.

Variables	Category	Crude OR (95% CI)	Adjusted OR* (95% CI)
AOC	Korean	1	1
	Immigrants	**0.679 (**0.611–0.755)	**0.761 (**0.670–0.863)

*Adjusted for type of insurance, residential region, most visited type of clinic, comorbid psychiatric disease, number of household members, doctor specialty, and antidepressant prescribed at the first visit.

### Determinants of AOC in depression management

Socioeconomic determinants affecting depression management in immigrants were evaluated by multivariate logistic regression. Immigration status was not associated with AOC in depression management. Sex and CCI score were likewise not associated with AOC. Advanced age showed a trend to increase AOC (40- to 64-year group; aOR, 1.412, 95% CI: 1.096–1.821; ≥65-year group: aOR, 1.464, 95% CI: 0.988–2.169).

Medical Aid (aOR, 2.309; 95% CI, 1.479–3.610), rural residence (aOR, 1.536; 95% CI, 1.054–2.237), the presence of a psychiatric comorbidity (aOR, 1.912; 95% CI, 1.484–2.463), and visiting a psychiatrist (aOR, 2.387; 95% CI, 1.821–3.125) were associated with a higher likelihood of AOC.

In contrast, visiting a higher-level hospital showed a tendency to reduce the likelihood of AOC, with an aOR of 0.804 for visiting a secondary hospital (95% CI, 0.636–1.016, insignificant) and an aOR of 0.623 for visiting a tertiary hospital (95% CI, 0.414–0.919). Having a non-SSRI regimen (TCAs and others) was also associated with lower likelihood of AOC, with an aOR of 0.749 for TCA (95% CI, 0.552–1.016, insignificant) and an aOR of 0.863 for others (95% CI, 0.493–0.895) (Table 4).

**Table 4:** Factors affecting appropriateness of care (AOC) in depression management among immigrants by multivariate logistic regression.

Variables	Category	Odds Ratio	95% CI
Immigration status	Naturalized citizen	1	
	Marriage immigrant	1.159	0.867–1.550
	Permanent resident	1.109	0.842–1.504
Sex	Male	1.000	
	Female	1.001	0.750–1.337
Age	20–40	1.000	
	**40–64**	1.412	1.096–1.821
	≥65	1.464	0.988–2.169
CCI score	0	1.000	
	1	0.861	0.668–1.109
	≥2	1.117	0.814–1.534
Type of insurance	NHI	1.000	
	**Medical Aid**	2.309	1.479–3.610
Residential region	Urban site	1.000	
	**Rural site**	1.536	1.054–2.237
Most visited type of clinic	Clinic	1.000	
	Secondary hospital	0.804	0.636–1.016
	**Tertiary hospital**	0.623	0.414–0.919
Comorbid psychiatric disease*	No	1.000	
	**Yes**	1.912	1.484–2.463
Number of household members	1	1.000	
	2–3	1.276	0.991–1.642
	>4	1.259	0.925–1.712
Doctor specialty	Others	1.000	
	**Psychiatrist**	2.387	1.821–3.125
Antidepressant prescribed at the first visit**	SSRI	1.000	
	TCA	0.749	0.552–1.016
	SSRI-alfa^#^	1.328	0.935–1.887
	**Others**^##^	0.664	0.493–0.895

*Psychiatric comorbidities included: psychotic disorder, manic diseases, bipolar, other affective disorders, anxiety, eating disorder, sleep disorder, personality disorder, compulsive behavior disorder

**SSRIs are the standard primary treatment option for depression, and were therefore used as the reference category.

#SSRI plus other co-medications for depression treatment

##MAOIs (Monoamine oxidase inhibitors), SNRIs (serotonin-norepinephrine reuptake inhibitors), NDRIs (norepinephrine–dopamine reuptake inhibitors), NARIs (Noradrenergic reuptake inhibitors), NaSSAs (noradrenergic and specific serotonergic antidepressants), SARIs (Serotonin antagonist and reuptake inhibitors), etc.

Significant results are shown in bold type.

## Discussion

This is the first population-based study including all immigrants in Korea that assessed the characteristics and determinants associated with access to and utilization of depression care services. The Korean NHI program is a model in which the payments come from a government-run insurance body that all citizens pay into, prorated by income and assets. Since we utilized NHI claims data, which only include insurance-covered medical services, we included permanent residents, marriage immigrants, and naturalized immigrants who are eligible to receive benefits under the NHI program. Eligible subjects can receive the same medical services, as long as they pay their copayments and medical expenses.

Although its cause is unclear, MDD is about twice as common in women as in men [32], and the first depressive episode most often occurs between the ages of 30 and 40, with a second peak between ages 50 and 60 [33]. This study showed that 5 times more women than men had MDD among immigrants, with the highest prevalence in the 40- to 60-year age groups (Table 1). The high proportion of MDD among women might reflect the marriage immigration trend in Korea. The proportion of female immigrants was 84.6% in 2015 according to the 2016 Korea National Statistics [34].

Immigrants were 0.761 times less likely to achieve AOC (Table 3), with 8.7% fewer immigrants than native Koreans achieving AOC in the 1-year follow-up period (Table 2). The number of patients who achieved AOC was higher in the acute phase than in the continuation period both in immigrants and native Koreans [54% vs. 25.2% (immigrants), 56.8% vs. 33.7% (Koreans)], but the decrement was larger in immigrants [28.8% (immigrants) vs. 23.1% (Koreans)]. This result agrees with that of a Swedish study, which evaluated medical adherence in immigrants and concluded that immigrants from outside Europe had a lower prescription pick-up rate (a proxy for health care adherence and utilization) than native Swedes [35].

Increasing evidence shows that early interventions in young people with depression and first-onset psychosis can improve immediate and long-term health outcomes [36], as can early diagnosis and appropriate interventions in general mental health practice [37]. Additionally, in a previous study, non-remission status after 3 months of treatment (OR = 3.56, p = .003) and inadequacy of early pharmacotherapy (OR = 2.73, p = .009) strongly predicted major depression status at 6 months[38]. The National Institute for Health and Care Excellence (NICE) guideline suggests that adult patients should be followed up every 2 to 4 weeks for the first 3 months to ensure that their treatment is effective [39]. According to the 2008 American College of Psychiatry guideline, treatment for major depressive disorder should be modified if the patient does not respond adequately to antidepressants within 6–8 weeks. [40]. Therefore, the identification of immigrants who do not receive appropriate pharmacotherapy or who have not recovered by 3 months may provide further treatment strategies to optimize depression care [38].

When considering the predictors evaluated by multivariate logistic regression, Medical Aid status strongly increased the likelihood of AOC, by 2.309 times compared to NHI coverage, from which it may be inferred that economic factors are the strongest predictor of healthcare utilization (Table 4). This result conforms to the well-known finding that economic factors constrain healthcare utilization [35, 41].

Rural residence was associated with a greater frequency of AOC in this study, which is contrary to the general consensus that high healthcare accessibility improves healthcare utilization; our findings could be interpreted as resulting from a tendency for Medical Aid–eligible immigrants, who presented high rates of AOC, to reside in rural sites. In accordance with this possibility, Table 1 presents roughly the same proportions for Medical Aid coverage (6.7%) and rural residence (7.7%).

Sex did not show statistically significant effects on AOC, but older individuals tended to be more compliant with depression management (Table 4). Even though elderly patients have compliance problems due to immobility and cognitive dysfunction [42], it is also a well-known phenomenon that younger patients are less compliant in the care of their diseases than older patients.

Comorbid psychiatric disease was associated with higher health care utilization among immigrants, and treatment by a psychiatrist was more effective for maintaining depression management. A recent study conducted in a mid-Atlantic state in the US reported that psychiatrists were 1.5 times more likely to prescribe suitable antidepressants than non-psychiatric specialists [43]. Receiving care from a doctor specializing in the relevant disease seemed to make appropriate management more likely. It has been reported that up to 50% of depression patients are not well detected in primary care settings [44]. Therefore, to improve depression management in general, a more effective routine screening strategy for depression needs to be incorporated into primary care [45, 46].

Primary general clinics are easy to access and often used for early screening for mental disorders, especially for communities with a high prevalence of depression [47]. This tendency was confirmed in this study, since tertiary hospitals were associated with a low likelihood of AOC, meaning that immigrants were reluctant to go to tertiary hospitals for depression management. Therefore, primary care settings can be a good place for early screening, but patients should be referred for treatment to mental health specialists, who can provide an adequate medical evaluation, psychotherapy, and pharmacotherapy [48].

SSRI and SSRI-based complex regimens presented an increased likelihood of AOC, whereas TCAs and other types of medications showed a decreased likelihood of AOC (Table 4). This result conforms to previous evidence on standard pharmacotherapy; SSRIs have been proven to be the most suitable primary treatment option for depression management by a large body of placebo-controlled studies, and no other group of medication has shown greater efficacy than SSRIs [49–51].

Although AOC was less achievedin immigrant patients, the difference between immigrants and native Koreans was not especially large. This finding might be understood in the context of “stigma,” which we could not assess in this study. The culturally specific concept or presupposition that mental disorders may be unacceptable to society could shape the low rates of depression management in native Koreans [52]. This is a well-known reason for low healthcare-seeking behaviors in patients with depression, mood, and substance disorders reported by a World Health Organization survey of 17 countries [53]. A recent cross-sectional survey reported a similar result. The researchers compared the perception of mental health and mental health services between college students in Vietnam and the US, and concluded that the Vietnamese students tended to believe that mental disorders are not acceptable to the community, whereas the US students perceived that mental disorders are the same as other diseases. The Vietnamese students were less likely to seek formal help than to seek help from family or friends [54]. Since a large proportion of immigrants to Korea come from other Asian countries, we might speculate that similar cultural and social attitudes towards mental disorders among immigrants and native Korean resulted in relatively minor differences in healthcare-seeking behaviors for depression management. Additionally, the immigrants included in this study were eligible to receive coverage under the Korean NHI; the similarity in the health care options available to the immigrants and native Koreans in this study may also explain the small difference in AOC between the groups.

Dozens of Korean studies have examined the risk factors of depression, especially in female immigrants. Kim et al (2013) [13] reported that elevated acculturative stress and less life satisfaction were significant risk factors of depression prevalence in female marriage immigrants. Nho et al (2017) [55] conducted a meta-analysis of 25 articles regarding depression in female marriage immigrants. They suggested that socioeconomic variables (language barrier and economic hardship) had a major impact on depression, and that psychological variables (acculturative stress, social support, marital satisfaction, life satisfaction, and self-esteem) had a moderate impact on depression. However, no previous study has encompassed all immigrants in Korea and evaluated the determinants affecting their healthcare access and utilization.

Since more frequent clinical visits enhance adherence and help patients avoid demoralization before experiencing the onset of beneficial effects [56], maintaining regular clinical visits in the acute and continuation phases of depression management promotes AOC for depressed patients. Within this framework, this study analyzed the characteristics and determinants associated with depression management among all immigrants in Korea.

In light of the expanding number of immigrants and the increasing need to implement an equitable healthcare policy for immigrants, we identified some significant socioeconomic determinants to consider in future healthcare policy-making aimed at immigrants. Additionally, in the context of low healthcare-seeking behavior for mental disorders among both immigrants and native Koreans, we ascertained that providing more active and effective education on the benefits of early and consistent interventions for mental disorders is imperative in Korea.

A few limitations of this study should be mentioned. First, several studies that evaluated the factor of country of origin showed different patterns in depression incidence and healthcare-seeking behaviors according to country of origin. However, our data source did not contain the exact country of origin for most immigrants, so we could not evaluate the socioeconomic determinants associated with this factor. Second, since the immigration history of Korea only lasts roughly 20 years, we could not conduct a generation-based analysis, which some previous studies have pointed out as a predictor for depression incidence and management. Last, since the evaluation of AOC was simply based on the number of visits, our results cannot fully reflect the practical and clinical impact of depression management, both in immigrants and native Koreans. However, our study presents some important implications for developing promising health strategies aimed at immigrants.

## Conclusion

Determinants associated with improved healthcare utilization of depression management in immigrants were insurance type (Medical Aid), place of residence (rural site), psychiatric comorbidities (comorbid status), doctor specialty (psychiatrist) and SSRI-based treatment regimens. When developing and introducing a healthcare strategy for immigrants, these findings should be taken into account.
